# Association of Wrist Circumference and Waist-to-Height Ratio with Cardiometabolic Risk Factors among Type II Diabetics in a Ghanaian Population

**DOI:** 10.1155/2018/1838162

**Published:** 2018-02-19

**Authors:** Christian Obirikorang, Yaa Obirikorang, Emmanuel Acheampong, Enoch Odame Anto, Emmanuel Toboh, Evans Adu Asamoah, Bright Amakwaa, Emmanuella Nsenbah Batu, Peter Brenya

**Affiliations:** ^1^Department of Molecular Medicine, School of Medical Sciences, Kwame Nkrumah University of Science and Technology (KNUST), Kumasi, Ghana; ^2^Department of Nursing, Faculty of Health and Allied Sciences, Garden City University College (GCUC), Kenyasi, Kumasi, Ghana; ^3^School of Medical and Health Sciences, Edith Cowan University, Joondalup, WA, Australia; ^4^Diagnostic Unit, Dansoman Polyclinic, Ghana Health Service, Accra, Ghana; ^5^Department of Medical Laboratory Technology, Faculty of Allied Health Sciences, KNUST, Kumasi, Ghana

## Abstract

The study determined the association of wrist circumference (WrC) and waist-to-height ratio (WHtR) with cardiometabolic risk factors among diabetics in a Ghanaian population. This cross-sectional study involved 384 diabetic patients at Begoro District Hospital, Ghana. Blood pressure, anthropometrics, and biochemical indices were measured. The overall prevalence of dyslipidaemia, metabolic syndrome (MetS), and hypertension was 42.4%, 76.3%, and 39.8%, respectively. The optimum cut-off range of WrC to identify individuals at increased cardiometabolic risk was 17.5 to –17.8 cm for men and 16.0 to 16.7 cm for women while that of WHtR was 0.52 to 0.61 for men and 0.53 to 0.59 for women. WrC for women was a significant independent predictor for MetS [aOR = 3.0 (1.39–6.72), *p* = 0.005] and systolic blood pressure [aOR = 2.08 (1.17–3.68), *p* = 0.012]. WHtR was a significant positive predictor for triglycerides [aOR = 3.23 (0.10–3.82), *p* = 0.001] for women. Using Framingham risk scores, 61% of the subjects had elevated 10-year risk of developing cardiovascular diseases (CVDs), with no significant difference in gender prevalence. WrC [aOR = 6.13 (0.34–111.4), *p* = 0.107] and WHtR [aOR = 2.52 (0.42–15.02), *p* = 0.309] were associated with statistically insignificant increased odds of moderate-to-high risk of developing CVDs in 10 years. The use of gender-specific cut-offs for WrC and WHtR may offer putative markers for early identification of CRFs.

## 1. Introduction

Diabetes mellitus (DM) is one of the major public health problems worldwide, not only due to the increasing number of affected people, but also according to its relation with disability and premature mortality and not neglecting the costs involved in its treatment and prevention [1, 2]. Africa alone accounted for 14 million diagnosed cases of diabetes; as at 2015, Ghana recorded 266,200 cases with a prevalence rate of 1.9% in adults (20–79 years). About 4790 deaths recorded in the same year aside the many undiagnosed cases in Africa, type II diabetes mellitus (T2DM) accounted for 90–95% of all diagnosed cases [3, 4]. The relationship between obesity and diabetes has been well documented in the Ghanaian population [5, 6]. A study among urban and rural settlers in a Ghanaian population found an increased cardiometabolic risk factors among urban settlers due to their increased sedentary and unhealthy dietary habits [7]. In sub-Saharan Africa, hypertension followed by obesity was the commonly known cardiometabolic risk factors associated with the general adult population [8]. Obesity and overweight are associated with an increased cardiometabolic risk; however, this may vary significantly by age, dietary habits, gender, and even among participants with morbid obesity [9]. However, the argument about the most effective anthropometric index associated with cardiometabolic risk factors among diabetics remains unresolved. Cardiovascular disease (CVD) risk factors such as obesity, hypertension, and dyslipidaemia are common in patients with DM, placing them at increased risk for cardiac events [10, 11]. In addition, many studies have found biological mechanisms associated with DM that independently increase the risk of CVD in diabetic patients [10–12]. Body mass index (BMI) being the most studied anthropometric index has been reported to be significantly related to CVD risk factors as demonstrated by several prospective and cross-sectional studies [13–15]. However, there are cumulative doubts about its role in predicting CVD risk factors. This has led to an increasing evidence for abdominal obesity indices such as waist circumference (WC), waist-to-hip ratio (WHR), and waist-to-height ratio (WHtR) as predictors of CVD [16, 17]. In a study among South African blacks, WHtR was found as a significant predictor for all cardiometabolic risk components after 5 years in an adult population [18]. The application of WHtR provides an alternative anthropometric index of central obesity that avoids the limitations of WC because the inclusion of height into the index enhances the avoidance of any potential confounding of cardiometabolic risk by height [19]. Previous studies have found similar WHtR cut-offs for increased cardiometabolic risk among Caucasian and Asian populations as well as men and women [20]. WrC is a simple anthropometric tool for the measurement of skeletal frame size. It has recently been suggested in several studies to be associated with insulin resistance in obese children and adults [21, 22], its consideration as a measurement of peripheral fat distribution has attracted much attention [23]. Moreover, it is an easy tool to detect measures of skeletal frame size without being severely confounded by body fat variation [22].

Various other anthropometric indexes such as WC, hip circumference (HC), and WHR have been used to determine the index more closely related to cardiometabolic risk factors among diabetics. To our knowledge, no study has been done to determine the association of WrC and WHtR with cardiometabolic risk factors among diabetics in a Ghanaian population. In contrast to the routine management requirement, individual risk stratification is highly recommended, and there is the need to elucidate proper anthropometric measurement that defines individuals who are at risk of cardiometabolic complications. To fill this gap, the present study sought to determine the association between WrC and WHtR with cardiometabolic risk factors and ascertain WrC and WHtR predictive values for cardiovascular risk burden using FS10 lipid risk score system.

## 2. Methodology

### 2.1. Study Design and Setting

This was a cross-sectional study conducted among diabetic patients attending Begoro District Hospital diabetic clinic on a weekly basis. The diabetic clinic at Begoro District Hospital is the only hospital in Fanteakwa North District in the Eastern Region of Ghana that runs a diabetic clinic. Moreover, it is located in Begoro and records attendance of 110,134 people per annum from over 157 communities within the district. It also serves as the main referral facility for Community Health-based Planning Service (CHPS, i.e., a community health officer-led program widely implemented across Ghana, notable for its impact on under-5 and maternal mortality [24]) centers, Clinics and Health centers in the Fanteakwa District, and the adjoining districts. Therefore, sampling was without bias but with fair distribution covering the ethnic groups, the majority being Akans and Ga-dangmes within the district.

### 2.2. Study Population and Subject Selection

**Figure 1:**
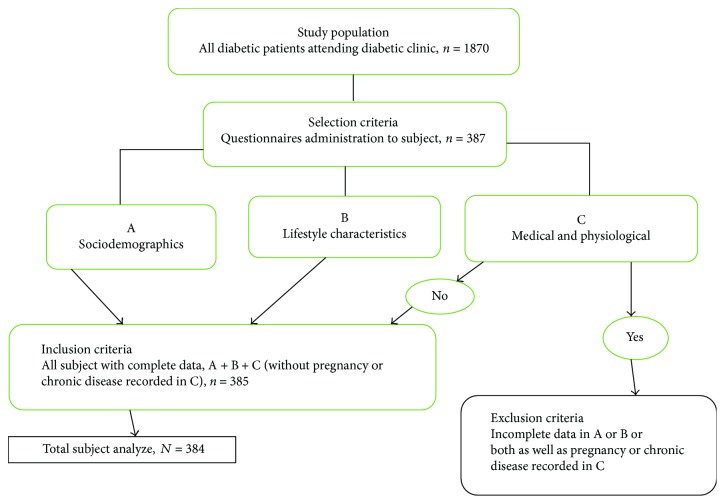
Flowchart of the protocol for the selection of subject.

Using a nonprobability convenience sampling, a total of 384 diabetics were recruited for the study. Selection of subject was done using a structured questionnaire. Trained nurses were placed at the Out-Patient Department (OPD) to administer the questionnaires regarding lifestyle (including smoking and drinking), medical history (including past illness history and medication history), physiological conditions (including pregnancy and fasting time), and sociodemographics, to all subjects during their health check-up. The researcher verified the completion of each questionnaire prior to collection. Subjects with incomplete data as well as those who were pregnant or had a chronic disease that may affect the metabolic status or body composition (e.g., thyroid or hypothalamic disease, chronic hepatitis, and cirrhosis) were excluded from the study. The sample used for the current analysis consisted of 147 men and 237 women. The protocol for the selection of subject is shown in Figure 1.

### 2.3. Inclusion and Exclusion Criteria

All diabetic individuals with complete data on sociodemographic and lifestyle characteristics without any chronic disease reviewed from their medical history were included in the study.

### 2.4. Sample Size Determination

A total of 384 diabetics were recruited from an estimated diabetic population of 1870, using a proportionate rate of 20.5%, confidence level of 95% (*z*-score 1.96), and margin of error of 5%. Using Cochran's formula [25], the minimum size required was 300; however, to accommodate a nonresponse rate of 10.0% and stronger statistical power and effect size, the samples were projected to 384 students.

### 2.5. Blood Pressure Measurement

Blood pressure (BP) was recorded after 5 minutes of rest with the subject being in the seated position using manual and an automated sphygmomanometer placed on the subject's right arm. This was measured three times, and the average reading was recorded. Individuals were deemed hypertensive if they were taking antihypertensive medications, if they self-reported a diagnosis of hypertension, if their systolic pressure was above 140 mmHg, if their diastolic pressure was above 90 mmHg, or a combination of these features.

### 2.6. Anthropometric Measurements

Body weight, expressed in 0.1 kg intervals, was measured at fasting state in the morning using automated scale. Portable height rod stadiometers were used for body height to the nearest centimeters. The subject stood straight, with feet placed together and flat on the ground, heels, buttocks, and scapulae against the vertical backboard, and arms loose and relaxed with the palms facing medially. Their heads were carefully positioned in the Frankfurt plane, with the lower margins of the orbit in the same horizontal plane as the upper margin of the external auditory meatus. BMI was calculated as body weight divided by height squared (kg/m^2^).

WrC was measured with subjects in a seated position using a tape measure positioned over the Lister tubercle of the distal radius and over the distal ulna. HC was measured at the level of maximal gluteal protrusion and waist circumference at the midpoint between the anterior superior iliac crest and the lowest rib using a tape measure while the subject stood with feet 25–30 cm apart. The tape measure was placed directly on the skin. Patients were allowed to breathe out normally, and measurements were taken. The tape was held lightly so as not to compress the skin. WHtR and WHR were calculated WC (cm) divided by Ht (cm) and HC, respectively. Body adiposity index (BAI) was calculated as the size of the hips compared to the patient's height [26].

(1) $$\begin{array}{c} BAI=HC\;\left(cm\right)÷{\left[height\;\left(m\right)\right]}^{1.5}-18. \end{array}$$

The conicity index (CI) was determined from the measurements of weight, height, and waist circumference [27].

(2) $$\begin{array}{c} Conicity\;index=\frac{WC\;\left(m\right)}{0.109\times \sqrt{weight\;\left(kg\right)/height\;\left(m\right)}}. \end{array}$$

Abdominal volume index (AVI) was derived from the measurements of waist circumference (WC) and hip circumferences (HC) [28].

(3) $$\begin{array}{c} AVI=\frac{2{\left[WC\;\left(cm\right)\right]}^{2}+0.7{\left[WC\;\left(cm\right)-HC\;\left(cm\right)\right]}^{2}}{1000}. \end{array}$$

Visceral adiposity index (VAI) uses the study participant's waist circumference (WC), BMI, triglyceride (TG), and HDL-C levels [29].

(4) $$\begin{array}{c} VAI\text{ for }females=\frac{WC}{36.58}+\left(1.89\times BMI\right)\times \left(\frac{TG}{0.81}\right)\times \left(\frac{1.52}{HDL}\right),\\ VAI\text{ for }males=\frac{WC}{39.68}+\left(1.88\times BMI\right)\times \left(\frac{TG}{1.03}\right)\times \left(\frac{1.31}{HDL}\right). \end{array}$$

All measurement was done by two (2) health technicians, one being the examiner and the other being the recorder.

### 2.7. Biochemical Analysis

Subjects fasted for a minimum of 12 hours and avoided a high-fat diet and alcohol consumption for at least 24 h prior to phlebotomy. Fasting blood glucose was measured using the One Touch glucometer and recorded. A fasting venous blood sample was obtained between 6:00 am and 11:00 am into a gel separator tube; sample allowed to clot and centrifuged at 5000 rpm room temperature for 5 minutes. Serum was then separated into a plain tube and stored in a 4°C refrigerator prior to analysis in the hospital laboratory. Clinical chemistry workup included total cholesterol (TC), high-density lipoprotein cholesterol (HDL-C), very-low-density lipoprotein (VLDL-C), low-density lipoprotein (LDL-C), and triglycerides (TG) using the COBAS INTEGRA® 400 plus automated chemistry analyzer. Other parameters like the coronary risk were calculated by dividing TC by HDL-C. A diagnosis of MetS was defined as central obesity plus either dyslipidaemia and hypertension or raised plasma glucose as illustrated in Table 1. Cardiometabolic risk was defined as a cluster of hypertension, metabolic syndrome, and an enhanced waist circumference (above 102 cm in males and 88 cm in females) accompanied by the alterations in lipid profile quoted above (HDL cholesterol below <1.03 mmol/L in males and <1.29 mmol/L in females, and serum triglycerides above ≥1.7 mmol/L).

### 2.8. Statistical Analysis

All statistical analyses were performed separately according to sex by using the Statistical Package for Social Science (SPSS version 12.0). The basic characteristics were presented by descriptive analysis as means and standard deviations (SD) for continuous data and as frequencies for categorical data. Comparisons between men and women were made using independent samples *t*-tests for continuous data and chi-square tests for categorical data. Pearson's correlation coefficients were used to determine the correlation between anthropometric indices and cardiometabolic risk factors. Receiver operating characteristic (ROC) curves were used to demonstrate the discriminatory ability of an anthropometric index over the entire range of possible values in the detection of a cardiometabolic outcome as quantified by the area under the curve (AUC). The optimal cut-off point for each anthropometric variable in the prediction of a given cardiometabolic outcome was established based on the highest combination of sensitivity and specificity. *p* values of less than 0.05 were considered to indicate statistical significance.

## 3. Ethical Consideration

The investigations were approved by the Committee on Human Research Publications and Ethics (CHRPE) at School of Medical Sciences, Kwame Nkrumah University of Science and Technology (KNUST), Kumasi, Ghana, in collaboration with the management of Begoro District Hospital. Participation was voluntary, and written informed consent was obtained from each participant according to Helsinki Declaration. Respondents were assured that the information gathered was to be used strictly for research and academic purpose only. In addition, respondents were given the freedom to opt out at any time they thought they could not continue with the study.

## 4. Results

The basic characteristics and prevalence of cardiometabolic risk factors of the study sample are presented in Table 2. The results found that mean age of men was significantly higher compared to the age of the women (60.8 ± 11.5 versus 55.0 ± 13.3, *p* = 0.029). Male participant had significantly larger wrist size than females (17.4 ± 0.8 versus 16.4 ± 1.0, *p* value < 0.0001). The variation was also seen among men and women where mean value for central adiposity (WHR) was significantly higher among men (0.96 ± 0.07 versus 0.91 ± 0.06, *p* value = 0.002). However, significantly higher TC and LDL-C levels were recorded in women compared with men (7.2 ± 1.4 versus 5.9 ± 1.6 and 5.15 ± 1.35 versus 3.89 ± 1.42, respectively, *p* value < 0.0001). BAI was also significantly higher in women compared to men (32.8 ± 6.2 versus 27.6 ± 3.6, *p* value < 0.0001). The study population was comparable in terms of cardiometabolic risk prevalence and anthropometric parameters other than the variations noted above (all *p* values > 0.05).

Table 3 illustrates the regression analysis of wrist circumference for dyslipidaemia and haemodynamic parameters. Regarding women, WrC was observed to be the significant positive predictors for MetS [aOR = 3.0 (1.39–6.72), *p* = 0.005] and SBP [aOR = 2.08 (1.17–3.68), *p* = 0.012]. The coefficients of determination were 0.221 and 0.481, which means WrC was significantly responsible for 22.1% and 48.1% for MetS and SBP. However, WrC was observed not to be statistically significant predictors for dyslipidaemia and haemodynamic indices in relation to men. WHtR was observed to be the significant positive predictors for TG [aOR = 3.23 (0.10–3.82), *p* = 0.001] for women not men.

Table 4 depicts the AUC of each anthropometric index in the prediction of multiple cardiometabolic risk among men and women. Each index apart from visceral adiposity index (VAI) was at its best in the prediction of MetS in both sexes ranging from 0.68 to 1.00 at 95% confidence interval, *p* values < 0.05. Among men and women, VAI was the best predictor of dyslipidaemia (TG and low HDL-C). The AUC of BAI in the prediction of TC was only significant in male subjects. However, the AUC of WrC, WHtR, AVI, VAI, CI, BAI, and BMI were best predictors of multiple cardiometabolic risk factors (hypertension, MetS, and dyslipidaemia) among male and female subjects, *p* values < 0.05. The cut-off value of WrC predictive of cardiometabolic risk factors was higher in male compared to that of female (17.5 to 17.8 versus 16.0 to 16.7). However, that of BAI was higher in women (30.5 to 32.8) than in men (24.2 to 29.5) in predicting cardiometabolic risk factors.  Table 5 presents the cut-off values, sensitivities, and specificities of anthropometric indices predictive of cardiometabolic risk factors.

Multiple logistic analyses of lipid profile, haemodynamic indices, and anthropometric indices for predicting incidence of 10-year CVD risk level among diabetic patients after controlling for age duration of diabetes and history of hypertension are shown in Tables 5 and 6. Subjects with poor controlled of FBS are significantly more likely to develop CVD [aOR = 5.0 (1.09–22.82), *p* = 0.038]. Similarly, increased likelihood was observed among patients with high TC [aOR = 2.65 (0.51–13.83)], TG [aOR = 2.26 (0.41–12.28)], LDL-C [aOR = 2.0 (0.46–8.72)], and MetS [aOR = 3.69 (0.98–13.86)] but was statistically not significant (*p* > 0.05). The presence of MetS [aOR = 4.53 (1.14–17.99), *p* = 0.031] and diagnosed as hypertensive using SBP [aOR = 68.2 (3.41–136.50), *p* = 0.0002] were associated with significant increased risk of CVD.

As shown in Table 7, larger wrist circumference was associated with increased odds [aOR = 6.13 (0.34–111.4)] of developing CVD in 10 years but was statistically not significant (*p* > 0.05). Similarly, increased odds were observed in other anthropometric indices for both moderated-to-high risk of 10-year incidence of CVD but did not show any significant results (*p* > 0.05).

## 5. Discussion

Cardiometabolic risk has commonly been used to describe the aggregate risk of developing cardiovascular disease. Although there is a general agreement upon such a risk, differences in the diagnosis of central obesity still exist [30]. In this study, in addition to conventional indices of central obesity, the recently introduced WrC and WHtR were included.

In this present study, WrC was significantly associated with MetS and hypertension (using SBP > 140) in diabetic female subjects, but no significant association was found in male subject. Moreover, WrC was a significant predictor of MetS and hypertension among female subjects even after controlling for subject's age, duration of diabetes, history of hypertension, and FBS (glycaemic control) (Table 3). This is inconsistent with the findings from a cross-sectional study by Jahangiri et al. [21] among Iranian population. The study found a significant positive association between WrC and MetS in both genders. In women population, WrC is independent from general and central obesity measures in the prediction of cardiometabolic abnormalities [16]. This could be expounded by the presence of “bone-fat-pancreas” axis that regulates energy hemostasis and coordinates energy partitioning between bone and adipose tissue and impacts insulin sensitivity [23]. In another cross-sectional study by Capizzi et al. [22] conducted among overweight and obese children and adolescents to identify WrC as a marker for insulin resistance, they found a significant positive association between WrC, its bone component, and insulin resistance, which is similar to our findings. Several other studies have also presented results corroborating our findings in this present study [23, 31]. WHtR was observed to be the significant positive predictors for TG for women not men in this study, which is in concordance with the findings from previous studies [18, 32, 33].

Differences between the two genders regarding the association between wrist circumference and cardiometabolic risk factors occurrence could be due to the effects of sex steroid hormones and their interaction with bone metabolism and glucose homeostasis [21]. In a meta-analysis conducted by Ma et al. [34] to evaluate association between bone mineral density (BMD) and type 2 diabetes mellitus, they concluded that overall individuals with T2DM have about 25–50% SD higher BMD compared to nondiabetic control subjects. Physiologically, insulin has an anabolic effect on bone due to its structural homology to IGF-1 by interacting with the IGF-1 receptor, which is present on osteoblasts [34].

The current study identified the gender-stratified cut-off points of potent anthropometric indices that proved to be better predictors of cardiometabolic risk factors. The optimal cut-off value of WrC to identify individuals at increased cardiometabolic risk is between 17.5 and 17.8 cm for men and 16.0 and 16.7 cm for women while that of WHtR was between 0.52 and 0.61 for men and 0.53 and 0.59 for women. Consistency in risk estimation using established cut-off was poor to fair for WrC estimation; high systolic pressure, kappa (0.05) for women; and metabolic syndrome, kappa (0.23) for women and (0.32) for men. Consistency with WHtR was good for SBP, kappa (0.24 and 0.40) for men and women, respectively, to very good for metabolic syndrome, kappa (0.58) for men and (0.84) for women, respectively. The current study reported a 10-year risk of developing CVD among diabetes patients and the plausible risk factors. Using the Framingham risk scores, 61% of the subjects had an elevated 10-year risk of developing cardiovascular diseases (CVDs). Both male and female subjects showed descry prevalence of prejudicial cardiovascular risk and moderate to higher risk of developing CVD in the future. Cohering the results of this present study in part is a multicenter study in Brazil among patients with type II diabetes by Gomes et al. [35]. In another study among diabetic patients in Manipur, Northeast India, Tungdim et al. [36] found similar results that corroborate our present finding. Several other studies have reported a higher risk of CVD among women. Accumulating evidence from recent findings has demonstrated alterations in estrogen-related protective mechanism by diabetes [21, 37]. This poses adverse changes in cardiovascular risk leading to enhanced atherogenesis in females [36]. In a meta-analysis by Agatisa et al. [38] and Kanaya et al. [39] among T2DM patients, they also reported that although the odds of CVD mortality were higher among women than men, the number of excess deaths attributable to diabetes was higher among men after controlling for CVD risk. The high incidence of moderate-to-high incidence of 10-year cardiovascular among subjects could be explained in terms of their lifestyle without exercise, poor glycaemic control (74.6%), prevalence of hypertension (42.4%), high LDL (80.5%), and MetS (76.3%).

Literature has provided several contradicting findings concerned with detrimental effect of hyperglycaemia on cardiovascular risk profile. High blood glucose level leads to oxidative stress and mitochondrial overproduction of superoxide, which have been acknowledged in the pathogenesis of diabetic micro- and macrovascular complications [40]. Regular physical exercise has been reported to be associated with lower risk of cardiovascular morbidity and mortality, both in the primary and secondary prevention [41], while elevation of small dense LDL particles and ApoB in type II diabetes mellitus (T2DM) patients is a predictor of cardiovascular risk [42]. The results of this present study found that larger WrC was associated with increased odds of moderate and high risk of developing CVD in 10 years (Table 5). In a cohort study by Mohebi et al. [23], they reported that WrC was independently associated with hypertension and CVD outcome. WHtR was also a plausible risk marker for CVD events with increasing odds of moderate and high 10-year CVD incidence (Table 6). The WrC was assumed to be a possible surrogate of body bone status and did not measure any bone status measures; thus, we could not evaluate the true predictive power of bone status in relation to CVD outcomes. Notwithstanding that findings in this study are comparable to reports from previous studies, the sample size was small, and the use of nonprobability sampling approach may have affected the statistical power and introduced sampling bias.

## 6. Conclusion

Using gender-specific cut-offs for WrC may offer putative marker for early detection of cardiometabolic risk factors among diabetic patients; therefore, being a simple and easy-to-detect measure, WrC and WHtR could be regarded as a new anthropometric assessment for prediction of cardiovascular and metabolic complications. Moreover, considering central and general obesity measurements, increasing wrist circumference highlighted an independent risk for incident cardiometabolic risk factors only among diabetic women. Further study needs to be done to include measurements of bone status and body fat stores to evaluate the true predictive power of bone status in relation to cardiometabolic risk outcome in a larger population.

## Figures and Tables

**Table 1:** IDF definition of metabolic syndrome (IDF, 2006).

According to IDF, for a person to be defined as having cardiometabolic risk, the person must have a WC of >90 cm for men and >80 cm for women or BMI > 30 kg/m^2^ and any two of the following.		
Dyslipidaemia	Raised triglyceride, TG	≥1.7 mmol/L or specific treatment for this lipid abnormality
	Reduced HDL-C	<1.03 mmol/L in males <1.29 mmol/L in females or specific treatment for this lipid abnormality

Hypertension	Systolic blood pressure	≥130 mmHg
	Diastolic blood pressure	≥85 mmHg or treatment of previously diagnosed hypertension

Raised fasting plasma glucose	(FPG) ≥ 5.6 mmol/L or previously diagnosed diabetes	

**Table 2:** Descriptive of anthropometric indices, haemodynamic indices, and lipid parameters of study participants.

Variables	Total (*n* = 384)	Men (*n* = 147)	Women (*n* = 237)	*p* value
Age (years)	56.4 ± 13.1	60.8 ± 11.5	55.0 ± 13.3	**0.029**
Duration of diabetes (years)	9.3 ± 7.5	10.9 ± 8.0	8.8 ± 7.3	0.228
WrC (cm)^a^	16.5 ± 1.1	17.4 ± 0.8	16.4 ± 1.0	**<0.0001**
WC (cm)^a^	91.9 ± 10.7	92.4 ± 10.2	91.8 ± 10.9	0.794
WHR^a^	0.9 ± 0.1	1.0 ± 0.1	0.9 ± 0.1	**0.002**
WHtR^a^	0.6 ± 0.1	0.6 ± 0.1	0.6 ± 0.1	0.152
BMI^a^	26.2 ± 4.9	24.9 ± 3.4	26.6 ± 5.3	**0.047**
VAI^a^	3.4 ± 4.0	2.9 ± 2.9	3.6 ± 4.2	0.320
AVI^a^	17.2 ± 3.9	17.3 ± 3.7	17.2 ± 3.9	0.877
BAI (%)^a^	31.5 ± 6.1	27.6 ± 3.6	32.8 ± 6.2	**<0001**
CI^a^	1.3 ± 0.1	1.32 ± 0.1	1.30 ± 0.1	0.309
SBP (mmHg)^a^	133.3 ± 19.7	135.7 ± 16.2	132.6 ± 20.7	0.404
DBP (mmHg)^a^	82.0 ± 16.2	85.7 ± 10.0	80.8 ± 9.9	0.065
FBS (mmol/L)^a^	10.5 ± 4.2	10.8 ± 4.2	10.4 ± 4.2	0.700
TC (mmol/L)^a^	6.9 ± 1.6	5.9 ± 1.6	7.2 ± 1.4	**<0.0001**
TG (mmol/L)^a^	1.9 ± 0.9	1.9 ± 0.9	1.8 ± 0.9	0.972
HDL-C (mmol/L)^a^	1.2 ± 0.4	1.1 ± 0.4	1.3 ± 0.4	0.071
LDL-C (mmol/L)^a^	4.9 ± 1.5	3.9 ± 1.4	5.1 ± 1.4	**<0.0001**
VLDL (mmol/L)^a^	0.9 ± 0.4	0.8 ± 0.4	0.8 ± 0.4	0.972
Coronary risk^a^	6.1 ± 2.7	5.8 ± 3.1	6.1 ± 2.5	0.591
High SBP, *n* (%)^b^	260 (67.8)	110 (75.0)	155 (65.6)	0.350
High DBP, *n* (%)^b^	153 (39.8)	68 (46.4)	89 (37.8)	0.414
High TG, *n* (%)^b^	178 (46.4)	68 (46.4)	110 (46.7)	0.982
Low HDL, *n* (%)^b^	205 (53.4)	58 (39.3)	137 (57.8)	0.087
MetS IDF, *n* (%)^b^	293 (76.3)	99 (64.3)	189 (80.0)	0.088
MetS WHO, *n* (%)^b^	289 (75.4)	101 (75.0)	179 (75.6)	0.952
MetS AACE, *n* (%)^b^	303 (78.8)	116 (78.6)	187 (78.9)	0.917
MetS NHLBI, *n* (%)^b^	296 (77.1)	105 (71.4)	187 (78.9)	0.412
MetS NCEP ATP III, *n* (%)^b^	296 (77.1)	105 (71.4)	187 (78.9)	0.412

All numbers are means ± standard deviation unless specified. ^a^*t*-tests. ^b^Chi-square tests. BMI: body mass index; WC: waist circumference; WHtR: waist-to-height ratio; WHR: waist-to-hip ratio; BAI: body adiposity index; VAI: visceral adiposity index; CI: conicity index; AVI: abdominal volume index; MetS: metabolic syndrome; FBG: fasting blood sugar; SBP: systolic blood pressure; DBP: diastolic blood pressure; TC: total cholesterol; TG: triglycerides; HDL-C: high-density lipoprotein cholesterol. *p* < 0.05 = statistically significant.

**Table 3:** Cross-sectional association and predictability of baseline wrist circumference (WrC) for dyslipidaemia and haemodynamic indices.

Variable	Men				Women			
	*β* (SE)	*R* ^2^	aOR (95% CI)	*p* value	*β* (SE)	*R* ^2^	aOR (95% CI)	*p* value
	WrC							
MetS^∗^	0.80 (0.67)	0.45	2.23 (0.60–8.28)	0.230	1.118 (0.402)	0.221	3.06 (1.39–6.72)	**0.005**
TC^ɵ^	0.02 (0.54)	0.18	1.02 (0.35–2.95)	0.969	0.355 (0.424)	0.143	1.43 (0.63–3.28)	0.403
TG^ɵ^	0.24 (0.54)	0.06	0.98 (0.36–2.68)	0.963	0.021 (0.210)	0.01	1.02 (0.67–1.54)	0.919
HDL-C^ɵ^	1.42 (0.81)	0.29	4.14 (0.85–20.24)	0.079	0.201 (0.239)	0.027	1.22 (0.77–1.95)	0.401
CR^∗^	0.78 (0.72)	0.22	2.19 (0.54–8.90)	0.273	0.148 (0.217)	0.051	1.16 (0.76–1.77)	0.495
SBP^∗^	0.42 (0.54)	0.09	1.52 (0.52–4.38)	0.436	0.730 (0.292)	0.481	2.08 (1.17–3.68)	**0.012**
DBP^∗^	1.31 (0.71)	0.24	0.27 (0.07–1.08)	0.064	0.360 (0.244)	0.061	1.42 (0.89–2.31)	0.140
	WHtR							
MetS^∗^	**0.53 (0.81)**	**0.08**	**1.70 (0.40–2.01)**	**0.283**	**0.014 (0.102)**	**0.091**	**1.01 (0.43–1.07)**	**0.381**
TC^ɵ^	**0.17 (0.90)**	**0.13**	**1.19 (0.30–2.91)**	**0.558**	**0.243 (0.183)**	**0.074**	**1.28 (0.39–2.18)**	**0.459**
TG^ɵ^	**0.43 (0.81)**	**0.08**	**1.14 (0.31–2.22)**	**0.601**	**1.173 (0.228)**	**0.375**	**3.23 (0.10–3.82)**	**0.001**
HDL-C^ɵ^	**−0.30 (0.81)**	**0.21**	**0.74 (0.20–8.34)**	**0.815**	**0.293 (0.197)**	**0.058**	**1.34 (0.05–1.99)**	**0.431**
CR^∗^	**0.37 (0.75)**	**0.30**	**1.45 (0.17–2.83)**	**0.339**	**0.153 (0.216)**	**0.092**	**1.17 (0.53–1.95)**	**0.503**
SBP^∗^	**0.31 (0.54)**	**0.05**	**1.36 (0.12–2.19)**	**0.407**	**0.527 (0.191)**	**0.329**	**1.69 (0.51–2.25)**	**0.425**
DBP^∗^	**0.03 (0.21)**	**0.02**	**1.03 (0.18–1.25)**	**0.591**	**0.278 (0.37)**	**0.101**	**1.32 (0.51–1.72)**	**0.595**

^∗^Adjusted for fasting blood glucose, duration of diabetes, history of hypertension, and age. ^ɵ^Adjusted for fasting blood glucose, duration of diabetes, and age. CR: coronary risk.

**(a):** 

Risk factor	WrC	VAI	WHR	WHtR
*Men (n* = 147)				
Hypertension	**0.59 (0.41–0.77)** ^**∗**^	0.59 (0.37–0.81)	0.55 (0.34–0.76)	**0.67 (0.47–0.86)** ^∗^
Mets IDF	**0.69 (0.55–0.84)** ^∗^	0.59 (0.39–0.79)	**0.85 (0.72–0.98)** ^∗^	**0.99 (0.99–0.99)** ^∗^
High TC	0.57 (0.24–0.89)	0.50 (0.24–0.76)	0.54 (0.28–0.80)	0.57 (0.29–0.85)
High TG	0.59 (0.39–0.78)	**0.94 (0.90–0.98)** ^∗^	**0.70 (0.49–0.90)** ^∗^	0.53 (0.32–0.74)
Low HDL-C	**0.70 (0.56–0.84)** ^∗^	**0.95 (0.95–0.95)** ^∗^	0.50 (0.30–0.71)	0.57 (0.35–0.78)
*Women (n* = 237)				
Hypertension	**0.67 (0.67–0.67)** ^∗^	0.53 (0.41, 0.65)	0.59 (0.47–0.71)	**0.66 (0.57–0.79)** ^∗^
Mets IDF	**0.73 (0.66–0.81)** ^∗^	**0.71 (0.58–0.84)** ^∗^	**0.68 (0.52–0.83)** ^∗^	**0.87 (0.77–0.97)** ^∗^
High TC	0.54 (0.48–0.61)	0.56 (0.43–0.69)	0.54 (0.38–0.55)	0.57 (0.44–0.71)
High TG	0.52 (0.52–0.52)	**0.95 (0.92–0.99)** ^∗^	0.51 (0.38–0.63)	0.53 (0.41–0.65)
Low HDL-C	**0.60 (0.60–0.60)** ^∗^	**0.85 (0.78–0.93)** ^∗^	0.60 (0.48–0.71)	0.52 (0.40–0.64)

**(b):** 

Risk factor	AVI	CI	BAI	BMI
*Men (n* = 147)				
Hypertension	**0.66 (0.47–0.85)** ^∗^	**0.61 (0.41–0.82)** ^∗^	**0.72 (0.54–0.91)** ^∗^	**0.69 (0.50–0.88)** ^∗^
Mets IDF	**1.00 (1.0-1.0)** ^∗^	**1.0 (1.0-1.0)** ^∗^	**0.78 (0.62–0.94)** ^∗^	**0.81 (0.66–0.96)** ^∗^
High TC	0.53 (0.28–0.77)	0.51 (0.26–0.74)	**0.76 (0.54–0.98)** ^∗^	0.58 (0.32–0.84)
High TG	0.56 (0.35–0.77)	0.57 (0.37–0.78)	0.55 (0.34–0.77)	0.50 (0.29–0.72)
Low HDL-C	0.54 (0.33–0.75)	0.56 (0.35–0.76)	0.66 (0.45–0.86)	0.54 (0.33–0.75)
*Women (n* = 237)				
Hypertension	**0.66 (0.54–0.77)** ^∗^	**0.61 (0.49–0.73)** ^∗^	**0.63 (0.51–0.74)** ^∗^	**0.68 (0.57–0.79)** ^∗^
Mets IDF	**0.83 (0.71–0.96)** ^∗^	**0.68 (0.53–0.84)** ^∗^	**0.81 (0.69–0.94)** ^∗^	**0.88 (0.78–0.98)** ^∗^
High TC	0.58 (0.45–0.71)	0.59 (0.45–0.72)	0.57 (0.45–0.70)	0.58 (0.46–0.71)
High TG	0.54 (0.42–0.65)	0.52 (0.40–0.64)	0.58 (0.47–0.70)	0.56 (0.45–0.68)
Low HDL-C	0.50 (0.38–0.62)	0.51 (0.39–0.63)	0.51 (0.39–0.63)	0.50 (0.38–0.62)

Data are AUC (95% confidence interval). ∗ represents positive test if test variable < threshold. All values in bold are significant, *p* value <0.05.

**Table 5:** WrC and WHtR as a selected discriminator of cardiometabolic risk factors.

Discriminator	AUC	Cut-off	Sensitivity, %	Specificity, %	Kappa
*Women*					
WrC ∗ SBP	0.67	16.7 cm	43	84	0.05
WrC ∗ MetS	0.73	16.3 cm	61	72	0.23
WrC ∗ HDL-C	0.60	16.0 cm	39	82	0.04
WHtR ∗ SBP	0.67	0.55	80	53	0.24
WHtR ∗ MetS	0.99	0.53	90	78	0.58
*Men*				72	
WrC ∗ MetS	0.69	17.8 cm	50	90	0.315
WrC ∗ HDL-C	0.70	17.5 cm	55	77	0.18
WrC ∗ MetS	0.73	16.3 cm	61	74	—
WHtR ∗ SBP	0.66	0.61	39	100	0.402
WHtR ∗ MetS	0.87	0.52	100	90	0.837

**Table 6:** Multiple logistic analyses of lipid profile and haemodynamic indices for predicting incidence of 10-year CVD risk level among diabetic patients.

Variable	Moderate to high	
	aOR (95% CI)	*p* value
*TC*		
Low	1	
High	3.0 (0.65–13.74)	0.157
*TG*		
Low	1	
High	4.73 (0.93–23.82)	0.060
*HDL-C*		
High	1	
Low	2.0 (0.39–10.16)	0.067
*LDL-C*		
Low	1	
High	4.13 (0.99–17.28)	0.052
*CR*	1	
Normal	1	
Risk	3.78 (0.75–19.06)	0.107
*FBS*		
Poor control	2.79 (0.73–10.72)	**0.038**
Good control	1	
*SBP*		
Normal	1	
Prehypertension	2.71 (0.59–12.31)	0.196
Hypertension	68.2 (3.41–136.5)	**0.0002**
*MetS*		
No	1	
Yes	4.53 (1.14–17.99)	**0.031**
*Dyslipidaemia*		
No	1	
Yes	2.92 (0.69–12.19)	0.142
*Duration of disease*		
<5	1	
05–10	0.52 (0.12–2.35)	0.398
>10	3.68 (0.36–37.92)	0.273

aOR: adjusted odds ratio; CI: confidence interval; FBG: fasting blood sugar; SBP: systolic blood pressure; DBP: diastolic blood pressure; TC: total cholesterol; TG: triglycerides; HDL-C: high-density cholesterol; MetS: metabolic syndrome.

**Table 7:** Multiple logistic analyses of anthropometric indices for predicting the incidence of 10-year CVD risk level among diabetic patients.

Variables	Moderate to high	
	aOR (95% CI)	*p* value
*WrC*		
Normal	1	
Larger	6.13 (0.34–111.4)	0.107
*WC*		
Normal	1	
Risk	0.95 (0.18–4.94)	0.951
*BMI*		
Underweight	—	
Normal	1	
Overweight	0.97 (0.25–3.70)	0.959
Obese	5.41 (0.27–104.7)	0.302
*CI*		
Normal	1	
Risk	1.01 (0.26–3.90)	0.987
*VAI*		
Q1	1	
Q2	2.43 (0.39–15.27)	0.344
Q3	2.29 (0.36–14.43)	0.379
Q4	3.57 (0.58–22.02)	0.170
*WHR*		
Normal	1	
Overweight	1.33 (0.10–17.99)	0.827
Obese	1.19 (0.12–11.41)	0.880
*WHtR*		
Underweight	—	
Normal	1	
Overweight	3.67 (0.27–49.29)	0.327
Obese	2.52 (0.42–15.02)	0.309

aOR: adjusted odds ratio; CI: confidence interval; BMI: body mass index; WC: waist circumference; WHtR: waist-to-height ratio; WHR: waist-to-hip ratio; BAI: body adiposity index; VAI: visceral adiposity index; CI: conicity index; AVI: abdominal volume index.

## References

[1] World Health Organization (2015). *Global Status Report on Noncommunicable Diseases. Geneva: 2010*.

[2] World Health Organization (2009). *Global Health Risks: Mortality and Burden of Disease Attributable to Selected Major Risks*.

[3] IDF (2015). *Global Diabetes Scorecard: Tracking Progress for Action*.

[4] IDF (2013). *Global Diabetes Scoreboard: Ghana at a Glance*.

[5] Opare-Asamoah K., Majeed S., Quaye L. (2017). Assessing the prevalence of hypertension and obesity among diabetics in the Tamale Metropolis, Ghana. *British Journal of Medicine and Medical Research*.

[6] Obirikorang Y., Obirikorang C., Odame Anto E. (2016). Knowledge and lifestyle-associated prevalence of obesity among newly diagnosed type II diabetes mellitus patients attending diabetic clinic at Komfo Anokye Teaching Hospital, Kumasi, Ghana: a hospital-based cross-sectional study. *Journal of Diabetes Research*.

[7] Obirikorang C., Osakunor D. N. M., Anto E. O., Amponsah S. O., Adarkwa O. K. (2015). Obesity and cardio-metabolic risk factors in an urban and rural population in the Ashanti Region-Ghana: a comparative cross-sectional study. *PLoS One*.

[8] Kingue S., Rakotoarimanana S., Rabearivony N., Bompera F. L. (2017). Prevalence of selected cardiometabolic risk factors among adults in urban and semi-urban hospitals in four sub-Saharan African countries. *Cardiovascular Journal of Africa*.

[9] Nichols G. A., Horberg M., Koebnick C. (2017). Cardiometabolic risk factors among 1.3 million adults with overweight or obesity, but not diabetes, in 10 geographically diverse regions of the United States, 2012–2013. *Preventing Chronic Disease*.

[10] de Mattos Matheus A. S., Tannus L. R. M., Cobas R. A., Sousa Palma C. C., Negrato C. A., Gomes M. B. (2013). Impact of diabetes on cardiovascular disease: an update. *International Journal of Hypertension*.

[11] Rivellese A., Riccardi G., Vaccaro O. (2010). Cardiovascular risk in women with diabetes. *Nutrition, Metabolism and Cardiovascular Diseases*.

[12] Chyun D. A., Young L. H. (2006). Diabetes mellitus and cardiovascular disease. *Nursing Clinics of North America*.

[13] Monteiro C. A., Benicio M. D. A., Conde W., Popkin B. (2000). Shifting obesity trends in Brazil. *European Journal of Clinical Nutrition*.

[14] Yusuf S., Hawken S., Ôunpuu S. (2004). Effect of potentially modifiable risk factors associated with myocardial infarction in 52 countries (the INTERHEART study): case-control study. *The Lancet*.

[15] Pietrobelli A., Steinbeck K. (2004). Paediatric obesity: what do we know and are we doing the right thing?. *International Journal of Obesity*.

[16] Freedman D., Khan L., Serdula M., Dietz W., Srinivasan S., Berenson G. (2004). Inter-relationships among childhood BMI, childhood height, and adult obesity: the Bogalusa Heart Study. *International Journal of Obesity*.

[17] Ho S.-Y., Lam T.-H., Janus E. D. (2003). Waist to stature ratio is more strongly associated with cardiovascular risk factors than other simple anthropometric indices. *Annals of Epidemiology*.

[18] Ware L., Rennie K., Kruger H. S. (2014). Evaluation of waist-to-height ratio to predict 5 year cardiometabolic risk in sub-Saharan African adults. *Nutrition, Metabolism & Cardiovascular Diseases*.

[19] Sung R. Y., So H.-K., Choi K.-C. (2008). Waist circumference and waist-to-height ratio of Hong Kong Chinese children. *BMC Public Health*.

[20] Ashwell M., Hsieh S. D. (2005). Six reasons why the waist-to-height ratio is a rapid and effective global indicator for health risks of obesity and how its use could simplify the international public health message on obesity. *International Journal of Food Sciences and Nutrition*.

[21] Jahangiri Noudeh Y., Hadaegh F., Vatankhah N. (2013). Wrist circumference as a novel predictor of diabetes and prediabetes: results of cross-sectional and 8.8-year follow-up studies. *The Journal of Clinical Endocrinology & Metabolism*.

[22] Capizzi M., Leto G., Petrone A. (2011). Wrist circumference is a clinical marker of insulin resistance in overweight and obese children and adolescents. *Circulation*.

[23] Mohebi R., Mohebi A., Sheikholeslami F., Azizi F., Hadaegh F. (2014). Wrist circumference as a novel predictor of hypertension and cardiovascular disease: results of a decade follow up in a West Asian cohort. *Journal of the American Society of Hypertension*.

[24] Nyonator F. K., Awoonor-Williams J. K., Phillips J. F., Jones T. C., Miller R. A. (2005). The Ghana community-based health planning and services initiative for scaling up service delivery innovation. *Health Policy and Planning*.

[25] Cochran W. G. (1977). *Sampling Techniques-3*.

[26] Freedman D. S., Thornton J. C., Pi-Sunyer F. X. (2012). The body adiposity index (hip circumference ÷ height^1.5^) is not a more accurate measure of adiposity than is BMI, waist circumference, or hip circumference. *Obesity*.

[27] Shidfar F., Alborzi F., Salehi M., Nojomi M. (2012). Association of waist circumference, body mass index and conicity index with cardiovascular risk factors in postmenopausal women: cardiovascular topic. *Cardiovascular Journal of Africa*.

[28] Patil V. C., Parale G., Kulkarni P., Patil H. V. (2011). Relation of anthropometric variables to coronary artery disease risk factors. *Indian Journal of Endocrinology and Metabolism*.

[29] Amato M. C., Giordano C., Pitrone M., Galluzzo A. (2011). Cut-off points of the visceral adiposity index (VAI) identifying a visceral adipose dysfunction associated with cardiometabolic risk in a Caucasian Sicilian population. *Lipids in Health and Disease*.

[30] Numan Ahmad M., Halim Haddad F. (2015). Suitability of visceral adiposity index as a marker for cardiometabolic risks in Jordanian adults. *Nutricion Hospitalaria*.

[31] Amini A., Noureddin Soltanian B. I., Askari G. (2012). Association of wrist circumference with cardio metabolic risk factors. *Journal of the Pakistan Medical Association*.

[32] Miralles C. S. W., Wollinger L. M., Marin D., Genro J. P., Contini V., Dal Bosco S. M. (2015). Waist-to-height ratio (WHtR) and triglyceride to HDL-c ratio (TG/HDL-c) as predictors of cardiometabolic risk. *Nutricion Hospitalaria*.

[33] Ren X., Chen Z. A., Zheng S. (2016). Association between triglyceride to HDL-C ratio (TG/HDL-C) and insulin resistance in Chinese patients with newly diagnosed type 2 diabetes mellitus. *PLoS One*.

[34] Ma L., Oei L., Jiang L. (2012). Association between bone mineral density and type 2 diabetes mellitus: a meta-analysis of observational studies. *European Journal of Epidemiology*.

[35] Gomes M. B., Giannella-Neto D., Faria M. (2009). Estimating cardiovascular risk in patients with type 2 diabetes: a national multicenter study in Brazil. *Diabetology & Metabolic Syndrome*.

[36] Tungdim M. G., Ginzaniang T., Kabui G. P., Verma D., Kapoor S. (2014). Risk of cardiovascular disease among diabetic patients in Manipur, Northeast India. *Journal of Anthropology*.

[37] Lee W. L., Cheung A. M., Cape D., Zinman B. (2000). Impact of diabetes on coronary artery disease in women and men: a meta-analysis of prospective studies. *Diabetes Care*.

[38] Agatisa P. K., Ness R. B., Roberts J. M., Costantino J. P., Kuller L. H., McLaughlin M. K. (2004). Impairment of endothelial function in women with a history of preeclampsia: an indicator of cardiovascular risk. *American Journal of Physiology-Heart and Circulatory Physiology*.

[39] Kanaya A. M., Grady D., Barrett-Connor E. (2002). Explaining the sex difference in coronary heart disease mortality among patients with type 2 diabetes mellitus: a meta-analysis. *Archives of Internal Medicine*.

[40] Laakso M. (2010). Cardiovascular disease in type 2 diabetes from population to man to mechanisms. *Diabetes Care*.

[41] Tanasescu M., Leitzmann M. F., Rimm E. B., FB H. (2003). Physical activity in relation to cardiovascular disease and total mortality among men with type 2 diabetes. *Circulation*.

[42] Martín-Timón I., Sevillano-Collantes C., Segura-Galindo A., del Cañizo-Gómez F. J. (2014). Type 2 diabetes and cardiovascular disease: have all risk factors the same strength?. *World Journal of Diabetes*.

